# Can charcoal-based dentifrices change the color stability and roughness of bleached tooth enamel and resin composites?

**DOI:** 10.4317/jced.62319

**Published:** 2025-02-01

**Authors:** Murilo Guimarães Campolina, Priscila Agustinha Neves de Souza, Lia Dietrich, Carlos José Soares, Ceci Nunes Carvalho, Hugo Lemes Carlo, Gisele Rodrigues Silva

**Affiliations:** 1 Undergraduate student. Department of Operative Dentistry and Dental Materials, School of Dentistry, Federal University of Uberlândia, Uberlândia, Minas Gerais, Brazil; 2 Professor. Department of Operative Dentistry and Dental Materials, School of Dentistry, Federal University of dos Vales do Jequitinhonha e Mucuri, Minas Gerais, Brazil; 3 Professor. Department of Operative Dentistry and Dental Materials, School of Dentistry, Federal University of Uberlândia, Uberlândia, Minas Gerais, Brazil; 4 Professor. Dental School, CEUMA University, São Luis, Maranhão, Brazil

## Abstract

**Background:**

Given the increasing prevalence of individuals using charcoal-based dentifrices over extended periods without professional oversight, concerns have emerged regarding their potential effects. This study aimed to investigate the influence of different charcoal-based products on color change (ΔE00), gloss stability, and surface roughness of dental enamel (Intact or bleached) and resin composites subjected to 12-month simulated brushing.

**Material and Methods:**

132 bovine crowns were randomly allocated and restored with either SS-single-shade or conventional multi-shade, NH-nanohybrid, NF- nano-filled, or BF- bulk-fill composite. A conventional toothpaste (Control), a charcoal-based toothpaste (DC) , and a charcoal-based toothpowder (PO) were evaluated corresponding to 1-, 2-, 6-, and 12-month simulated tooth brushing, respectively. Color (ΔE00) and surface roughness (Ra) were assessed at baseline and after each brushing (n=11). Gloss (GU) was assessed before and after brushing. The brushing products, as well as the enamel and composite surfaces, were analyzed using scanning electron microscopy. Color change, gloss stability, and surface roughness were analyzed by a three-way repeated-measures analysis of variance followed by Tukey’s test (α=0.05).

**Results:**

Significant differences were found for composite color change (ΔE00) based on brushing product (*P*<0.001), brushing time (*P*<0.001), and their interaction (*P*<0.001), with similar changes across charcoal-based and conventional toothpaste. After 12 months of brushing with toothpowder (PO), all composites exceeded the acceptable ΔE00 threshold. Gloss values decreased significantly (*P*<0.001), with greater losses in NF and NH resins. Roughness (Ra) also increased more with PO than with toothpaste. Both PO and DC significantly reduced enamel gloss, especially on bleached enamel.

**Conclusions:**

Charcoal-based toothpaste alters the morphology, roughness, gloss, and color of enamel and resin composite, similar to regular toothpaste. Toothpowder creates surface irregularities, decreasing the gloss and increasing the color change and roughness, more strongly impacting bleached enamel and composites after 12-month simulated tooth brushing.

** Key words:**Activated charcoal toothpaste, roughness, gloss, color change, tooth abrasion.

## Introduction

Composite resins have grown in prominence as restorative materials since their advent, and are commonly employed to restore anterior (1) and posterior (2) teeth because of their excellent esthetics, reduced sound tissue removal, and acceptable longevity (1,2). The physical and mechanical properties of this restorative material and its cosmetic appearance have been improved through the development of matrix and filler technologies (3). To attain sufficient esthetics or provide speedy treatment for patients, different composites such as bulk-fill and single-shade became popular.

Single-shade restorative materials focus on color matching between materials and dental tissues, particularly in anterior restorations (3-6). The ability of these materials to blend colors is based on color shifting and increased translucency. In contrast to traditional composite systems based on several shades, single-shade composites were created to make restorative treatments easier by removing the color selection process and eliminating the need for many composites with various translucencies (3). However, color stability (7) is still an issue, despite breakthroughs in the quality of cosmetic restorative materials. Composite resins are prone to discoloration (7-9).

Bulk-fill composites focus on offering a time-saving treatment compared to traditional composites. The increased depth of cure and reduction of shrinkage stress, promote a streamline restorative procedure by requiring fewer increments to achieve a viable restoration (10). Since bulk-fill materials are highly translucent and allow light to pass through to permit the polymerization of the restoration’s deepest portions, they place less value on the material’s aesthetic attributes. The composite has a greyish tone as a result of the high translucency, which could be unsightly. (11) Even with those limitations, analyzing not just direct physical properties such as roughness but aesthetic color and gloss changes of bulk-fill composites could indicate impacts in the composite structure surface caused by the charcoal-based dentifrices, especially indicating abrasivity and strain that may cause degradation of the dental material.

The demand for esthetic dentistry has increased owing to the development of new restorative materials. The appearance of teeth is directly related to self-esteem and social interactions, and whiter teeth are desired by patients for aesthetic satisfaction. Recently, low-cost charcoal-based whitening toothpaste and toothpowder have become popular for oral hygiene (12-14) and promise to be helpful in treating discolored teeth without the need for a dentist’s supervision. Although these products are easily available through e-commerce sites, supermarkets, and pharmacies, there is limited evidence supporting their clinical benefits (13).

The composition (15), mode of production, and particle size distribution of charcoal used in the formulation are thought to affect the abrasives of charcoal-containing dentifrices (14-16). Charcoal in “charcoal toothpaste” is a fine powder form of activated charcoal that has been oxidized through controlled reheating or chemical processes (12,15). The more abrasive the formulation, the more efficient it will be at removing extrinsic stain and other tooth surface deposits. However, if the formulation is abrasive, it may result in a negative increase in the risk of superficial damage to dental tissues (14-19) or increasing color change and surface wear of tooth-colored restorations (8,15,20-25). However, time, brushing method, and brush features all affect the mode of action of activated-charcoal-based whitening dentifrices (25), just as they do with any other toothpaste (20). However, these dentifrices promote more optical and morphological changes on ceramic and conventional multi-shade composite surfaces (20).

Given the increasing number of people who use these products (13) for a long period without supervision, the ability of activated-charcoal-based dentifrices to produce dark gray foam during brushing, and the pigment absorption capacity of resin composites (15,20), the goal of this study was to investigate the effects of charcoal-based dentifrices on the color stability (26) and surface roughness of enamel and different resin composites submitted to 12-months of simulated brushing. The null hypotheses of this study were as follows: 1) charcoal-based dentifrices do not cause more composite color and gloss changes or roughness than those caused by conventional fluoride toothpaste after 12-month simulated brushing, and 2) color, gloss stability, and surface roughness do not differ between bleached or intact enamel brushed with conventional fluoride or charcoal-based products.

## Material and Methods

-Experimental design

**Figure 1:**
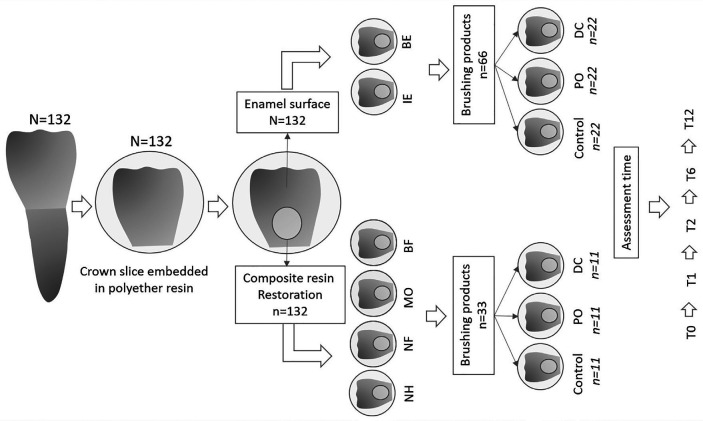
Schematic illustration of the experimental design.

The study design followed the CRIS (Checklist for Reporting *In vitro* Studies) tool guidelines, according to the recommendations for *in vitro* studies. One hundred and thirty-two bovine teeth originated from slaughterhouse donations, exempting them from requiring the ethical committee approval for animal testing. All tests followed the scientific requirements and research protocols established by the World Medical Association Declaration of Helsinki. The main outcome considered for determining the sample size was ΔE00, which considering the perceptibility color difference thresholds of 0.80 (7). The required sample size was calculated to be n = 11 per group, with a power test of 0.8, and α = 0.05, using the statistical software SigmaStat v.3.5 (Systat Software Inc., Chicago, IL, USA). Figure 1 shows a schematic of the experimental design.

-Specimen preparation

One hundred and thirty-two freshly extracted bovine incisors were obtained, cleaned, and stored in deionized water in a refrigerator at 4°C for no more than one month until the experimental stage. Double-faced diamond discs were used to remove the roots from the coronary sections (KG Sorensen, Barueri, Brazil).

The crowns were sectioned, and the pulp chamber was filled with nanohybrid composite resin (FGM, Dentscare LTDA, Joinville, SC, Brazil), and then embedded in polyether resin (Advanced Vacuum Trade-in Composite Materials LTDA, Santo André, SP, Brazil). The buccal surface was polished and standardized by wet grinding with #600-grit #1200-, #1500-, and #2000-grit silicon carbide papers (Wetordry 3M, Nova Veneza Sumaré, SP, Brazil) and alumina suspension at 3 µm (Buehler, Lake Bluff, IL, USA). The specimens were then numbered, randomly allocated, and subjected to different restorative materials (n=33) (Fig. 1).

A circular preparation 5 mm in diameter and 2 mm in depth was performed in the cervical portion of the crown using a wheel-shaped diamond drill (3053 – FG AllPrime, Imported by Dental Cremer, Blumenau, SC, Brazil) coupled in a cavity machine preparation. Then, enamel and dentin were prepared with 37% phosphoric acid (Ângelus Indústria de Produtos Odontológicas S/A, Londrina, PR, Brazil) for 30 and 15 s, respectively, washed with distilled water for 30 s and dried. Then, a single-bottle adhesive system (Ambar APS, FGM, Joinville, SC, Brazil) was applied and photoactivated for 20 s with a multiple peak light-curing device (Valo Cordless, Ultradent, South Jordan, Utah, USA), with an irradiance of 1000 mW/cm2 in standard mode. After inserting one of the tested composites into the prepared cavity (NH, NP, BF, or SS), a Myler strip was pressed over, using a glass slab to obtain a flat surface. The composite was photoactivated using the same photoactivation protocol described above, perpendicular and at the shortest possible standardized position between the tip and the tooth. The Myler strip and glass slab were removed, followed by additional light activation for 20 s. Then, after 24h, the specimens were polished with alumina suspensions at 6, 3, 1, and 1/4 µm (Buehler, Lake Bluff, IL, USA).

A half of the prepared teeth were subjected to enamel bleaching (27) sessions in a room at a controlled temperature (25.0 ± 1.0°C). The bleaching agent was applied according to the manufacturer’s instructions.  Table 1 shows the material used, its composition, and the manufacturer.

Restored teeth were divided into three groups (n = 11) according to the dentifrice: 1- conventional fluoride dentifrice with no charcoal- Control (Colgate Total 12, Colgate-Palmolive, New York City, NY, USA.), 2- charcoal-based toothpaste (DC- Colgate Natural Extracts) and 3- charcoal-based toothpowder (PO- Whitemax, Dermavita, Brusque, SC, Brazil).

The randomization process (blocked random scheme) was performed using www.sealedenvelope.com. The identification of the treatment to be applied to the samples followed the sequence numbered previously. Randomization and allocation were performed by the same researcher (GRS) who was not involved in the implementation (MGC) and evaluation process (PANS). Because they did not know the coding method, those who assessed the color and roughness measures (LD) and statically evaluated the data (GRS) were blinded.

-Brushing protocol

The specimens were subjected to mechanical brushing cycles in a toothbrushing machine (Odeme Dental Research, Luzerna, SC, Brazil), which was performed with a vertical loading of 300 g over the soft-bristle toothbrush heads (Colgate-Palmolive, São Paulo, SP, Brazil), temperature control (25 ± 1°C), and linear motion at 2 Hz (120 cycles/min). All specimens were immersed in a slurry prepared with toothpaste or charcoal powder, purified water, and artificial saliva in a 1:1:1 (w/v) proportion. Given that 73,000 mechanical brushing cycles correspond to five years of brushing exposure for a healthy person (21), the following brushing times were simulated: T1- 1-month, T2- 2-months, T6- 6-months, and T12- 12-months, with the specimens subjected to 1,217, 2,434, 7,300, and 14,600 brushing cycles, respectively. Every 300 cycles, the slurry created for each type of dentifrice is replaced. Before the brushing simulation, the baseline values (T0) for color measurements, gloss and roughness were calculated on composite and enamel surfaces, and the specimens were ultrasonically washed for 10 min and re-evaluated for roughness and color after each simulated brushing period. The samples were stored in artificial saliva (1.5 mM calcium [CaCl2], 0.9 mM phosphate [NaH2PO4], 0.15 mM KCl, pH 7.0; Pharmus, Uberlândia, MG, Brazil) at 4°C between assessment times.

-Surface roughness

At baseline (T0) and after each simulated period of tooth brushing (T1, T2, T6, and T12), the surface roughness was measured using a roughness meter (Mitutoyo, Aurora, IL, USA). The mean roughness (Ra) was measured with a static load of 5 N and speed of 0.05 mm/s, the mean roughness (Ra) was measured. In sequential mode, the cutoff value was 0.25 m, and the measurement distance was 1 mm. For each specimen, three readings were taken from the center of the surface of both the composite and the enamel, and the arithmetic mean was determined.

-Color analysis 

The color of the specimens was assessed using a visible/ultraviolet reflection spectrophotometer (Ci64UV, X-Rite, Grand Rapids, MI, USA). The device had an aperture diameter of 4 mm, and readings were performed with a 2° observer angle and illuminant D65. The coordinates of the LAB system from the Commission Internationale de L’Eclairage (CIE) were recorded. This system is based on lightness (coordinate L*) and chromaticity coordinates a* (red-green axis) and b* (yellow-blue axis). Color was measured at baseline (T0) and after T1, T2, T6, and T12 by placing the surfaces at the same position for all measurements on composite and enamel surfaces.

The color measurements were performed in triplicate on a white background (ColorChecker grayscale, X-Rite, Grand Rapids, MI, USA, L*white = 95.2, a*white = 21.2, b*white = 50.3), and the mean values were used for data analysis. The overall color changes from baseline compared with other assessment times (T1, T2, T6, and T12) were calculated using the CIEDE2000 color difference (∆E00), calculated as follows: ∆E00 = [(∆L/KLSL)2 + (∆C/KCSC)2 + (∆H/KHSH)2 + RT (∆C/KCSC) (∆H/KHSH)] 1/2, where ∆L, ∆C, and ∆H are considered lightness, chroma, and hue differences between color measurements. KL, KC, and KH are the parametric factors that influence the viewing and illuminating conditions. RT is the function of the hue and chroma interaction in the blue region. SL, SC, and SH are the weighting functions for color difference adjustment considering the location variation of the L*, a*, and b* coordinates, respectively (23).

-Gloss analysis (gloss units, GU) 

The gloss of the samples was measured using a glossmeter (3NH Global, NHG60M, Shenzhen, China) in gloss units (GU). An angle of 60° was applied to evaluate the gloss at the center of the sample on both composite and enamel surfaces. The mean of the three measurements per sample was calculated before and after the brushing procedure.

-Surface morphology

The morphology of the enamel and composite resin surfaces was examined before and after 12-months of simulated charcoal-based toothpowder brushing. The specimens were subjected to vacuum in a sputter coater (SCD 050 Sputter Coater, Capovani Brothers Inc., New York, USA) to deposit a thin layer of gold before being subjected to SEM (Zeiss EVO MA10, Jena, Thuringia, Germany) at 2000X magnification. Approximately 1 g of each toothpaste and toothpowder was dried at 37°C in an incubator for seven days. The dried materials were placed on stubs and sputter-coated with gold (SCD 050), -followed by SEM analysis at 2000X.

-Statistical analysis

Repeated-measures analysis of variance and Tukey’s tests were used to compare roughness, gloss, and color parameters (ΔE00) to both composites and enamel. The ‘brushing time’ was considered as a repetition factor. Statistical analysis was performed using the Jamovi statistical software package (version 2.0; dev.jamovi.org). The signiﬁcance level was set at α = 0.05 for all data analyses.

## Results

The mean and standard deviation of ΔE00 and gloss for the composite resins are listed in  Table 2. A significant difference was observed between the brushing product (*P*<0.001), brushing time (*P*<0.001), and interaction between both factors (*P*<0.001) for color change (ΔE00), regardless of the composite resin (*P*=0.156). The color change of the composites was similar when charcoal-based and conventional toothpaste were used. After toothpowder brushing (PO) to T2, the color shift for all the composites was considerable, but they reached values above the accepTable level (E00>1.77) (7) at T6. After brushing, all composites showed a decrease in gloss values (*P*<0.001). PO caused a greater loss of gloss in NF (*P*<0.001) and NH resins (*P*<0.001). However, charcoal-based products reduced the gloss similar to regular paste on SS (*P*=0.955) and BF (*P*=0.758) composite resin surfaces.

**Figure 2:**
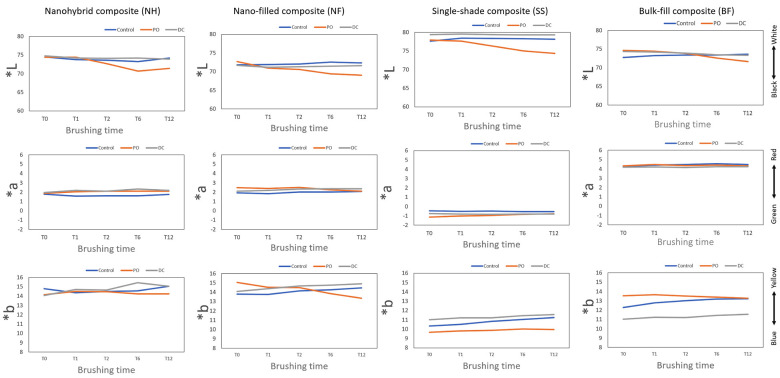
Graphs show the trends in the L*, a* and b*parameters of the composite resins over brushing time (L*: white/black; a*:red/green; b*: yellow/blue). Control: mechanical brushing with conventional fluoride dentifrice; PO: mechanical brushing with charcoal-based toothpowder; DC: mechanical brushing with charcoal-based toothpaste. “

Figure 2 shows the CIELAB parameters of the composite resins. L* is a parameter that usually represents the major concern from an aesthetic standpoint (darkness to lightness). All composite resins tended to decrease the L values using PO over the brushing time, losing whiteness.

**Figure 3:**
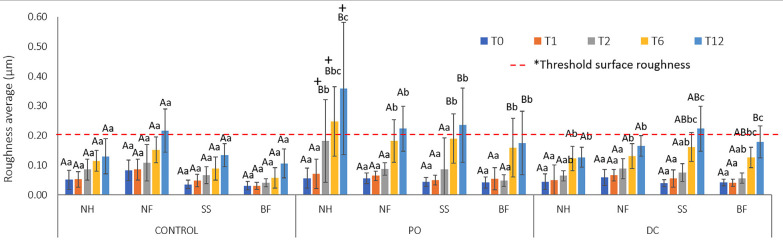
Means (±standard deviation) of composite roughness average (µm) after brushing products and times (n=11): T1- 1-month, T2- 2-months, T6- 6-months, and T12- 12-months. Different letters (lowercase for comparing the brushing times for composite*brushing product; uppercase for comparing brushing products for composite*time; symbol for comparing composites for brushing product*time) indicate significant difference at Tukey`s test (*P*<0.05). *Threshold roughness (19)”

The average mean and standard deviation of the composite roughness are presented in Figure 3. Significant differences were observed between the brushing product (*P*<0.001), brushing time (*P*<0.001), composite (*P*<0.001), interaction between time*product (*P*<0.001), composite*product (*P*<0.001), and time*brushing product*composite (*P*<0.001) for roughness (Ra). PO resulted in a higher Ra than toothpaste (control or DC) on the resin surface. The roughness increased at T2 for the nanohybrid and T6 for the other composites. Single-shade composites showed Ra values similar to those of conventional nano-filled and bulk-fill composites. After 12-months, NH, NF and SS composites brushed with charcoal toothpowder were above the Ra threshold of 0.2 µm (18).

Repeated measured ANOVA indicated no differences between groups for enamel ΔE00 (*p*=0.652) ( Table 3). However, after 12 months of simulated brushing, both PO and DC significantly reduced the gloss of the enamel compared to the control group. PO caused a more pronounced reduction in gloss on bleached enamel than on intact enamel. Similarly, there was an increase in the average roughness of the enamel, with a greater impact observed when using PO on bleached enamel ( Table 4).

**Figure 4:**
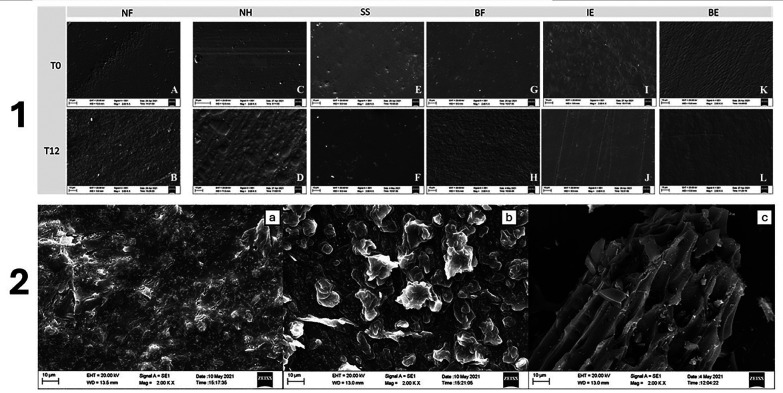
Scanning electron microscope (SEM) images. 1. Composite resins surfaces before (T0) and after 12-month simulated brushing (T12) at 2000X. NH. Conventional nanohybrid, A- (T0) and E- (T12); NF. Conventional nano-filled, B- (T0) and F- T12); SS. Single-shade, C- (T0) and G (T12); BF. Bulk-fill, D-(T0) and H- (T12). 2. Representative Scanning microscopy images of brushing products. a- Conventional fluoride dentifrice (Control - Colgate total 12, Colgate-Palmolive). b- Charcoal-based dentifrice (DC- Colgate Natural Extracts, Colgate-Palmolive). c- Charcoal-based toothpowder (PO- WhiteMax). “

Photomicrographs of the particles in the dentifrices are shown in Figure 4. The toothpastes (control and DC) showed irregularly shaped particles and spherical nanoparticle clusters. Abrasive particles with sharp angles, irregular appearance, and porosities were present in the PO, and they were larger than other dentifrices.

SEM images revealed surface alterations in all composites after 12-months of simulated brushing with toothpowder; however, NH had more irregularity than the other restorative materials (Fig. 4).

## Discussion

Composites provide easier esthetic performance during dental restorative procedures, resulting in a more natural-looking restoration (1-3). However, patients may attempt to improve their dental esthetics independently by purchasing and using products like charcoal-based whitening toothpaste and toothpowder, without professional guidance (13). The interaction between these products with enamel and composites is not well understood in the literature, and given the widespread availability of charcoal-based products, it was crucial to investigate this relationship.

According to the present study, charcoal-based toothpowder causes more composite color change and surface roughness with decreasing surface gloss than conventional or charcoal-based toothpastes after simulated brushing, which led to the rejection of the first null hypothesis. However, no differences were observed between the two types of toothpaste. This suggests that the charcoal-based toothpaste tested did not induce further color change of the composite surface compared to that produced by the mechanical and chemical action of the regular fluoride toothpaste. Thus, despite the fact that the dark color of the material is similar to that of other pigmenting agents, such as wine, black tea, or coffee (19), these toothpastes may not be linked to an increased risk of making the composite resin darker in color after 12 months of brushing. Conversely, a previous study (20) reported that charcoal-based toothpaste resulted in greater color change than conventional dentifrices. These contradictory results could be explained by the differences in the tested formulations and methodologies. Our samples had a lower baseline roughness, and ultrasonic vibration after brushing may have reduced the amount of charcoal stain remaining on the composite surface. As a result, the gray component of the charcoal-based toothpaste had less impact on the color change.

Considering dental enamel, the second null hypothesis was also rejected. Although charcoal powder does not significantly affect the color change of dental enamel after brushing—neither darkening nor lightening the teeth—it does reduce gloss and increase roughness, particularly on previously bleached enamel (27). This effect may be attributed to mineral structural changes in bleached enamel (27), making it less resistant to abrasive agents. Given that activated charcoal has abrasive properties, its use on bleached enamel can lead to more pronounced surface degradation, resulting in increased roughness and alterations in color and gloss.

Surface roughness increasing values can promote accelerated abrasion and reduce the resistance of enamel (17) and composite mechanical properties. No significant difference was found in composite surface roughness using toothpastes. The activated charcoal used in toothpaste is a fine powder that varies in abrasiveness according to its source, production method, particle size, shape, and hardness (14,15). When the SEM images were evaluated, a similar pattern of abrasive particles was observed between the toothpastes, justifying the similar behavior regarding abrasiveness and resulting in similar morphological patterns and surface roughness of the composites and enamel. In this context, a previous study also revealed that charcoal-based toothpastes do not necessarily cause more abrasion on enamel than conventional toothpastes (16). However, all the tested brushing products caused gloss values below those considered clinically acceptable (GU >40) for enamel resin composites, which indicate aesthetic problems (9). The capacity of a surface to reflect light is known as gloss and is correlated with surface smoothness, permitting a range of light reflection indices. (26)

Visual thresholds are important qualitative indicators in dentistry for evaluating and interpreting clinical outcomes and comparing treatments. After a two-month period, charcoal-powder brushing was able to cause higher color changes than toothpaste, reaching values above the clinical acceptability threshold (ΔE00>1.77) (7) at six months. Composite resins are restorative materials that are prone to color changes as the organic matrix absorbs water, making them more sensitive to staining agent penetration. All composites tended to exhibit decreased L values over brushing time, which indicates that the use of toothpowder for up to 1 month may lead to the need to replace the anterior restorations due to complaints about esthetics in the smile zone. Similarly, surface roughness increases for almost all composites (NH, NP and SS), being considered above threshold roughness value of 0.2 µm after six months (18). Rough surfaces result in the accumulation of plaques and stains, which may lead to discoloration and secondary caries. These data corroborate those of other studies that reported greater composite wear and tear with toothpowder (20).

SEM analyses showed that the toothpowder had larger and porous particles with angular shapes that visually differed from the spherical nanoparticles and nanoclusters present in both toothpastes. Consequently, the increased activated charcoal content, particle shape, and toothpowder size might have increased the roughness of the composite surfaces. Moreover, this finding could be explained not only by the lower abrasiveness of these toothpastes, but also by the inclusion of some compounds in their formulations, such as water, xanthan, cellulose-based gums, and carboxymethylcellulose, which can impact anti-erosive properties.

The current study’s findings confirmed that morphological composite surface changes are material- (18,24) and aging-time dependent (21). The resin matrix composition, matrix/particle interface, particle shape and size, degree of polymerization, and hardness of resin composites can all affect abrasion resistance (25). The conventional nanohybrid with a larger filler size presented an increase in the mean surface roughness after 2-months of simulated brushing with toothpowder. Ra values were twice the clinical threshold (18) at 12-months. An analysis of the SEM images revealed holes and scratches on the surface of the nanohybrid resin. Larger and more protruded filler particles indicate greater energy generated by abrasion processes, which is transmitted directly to the surrounding matrix, causing microcracks to propagate and cause particle detachment, thereby increasing the roughness and potentiating the restoration wear process (26). Surface roughness is important from a clinical standpoint. Surface roughness can enhance biofilm formation and buildup, causing irreversible damage to tooth hard tissues and restoration surfaces because it strongly impacts bacterial adherence (18). Surface roughness can also cause gingival recession, periodontal inflammation, dentin hypersensitivity, and the accumulation of oral pigments, all of which can affect the appearance of enamel and restoration margins (18).

Remarkably, despite the differences among the corresponding translucency, fillers, and matrix characteristics and compositions, similar outcomes were obtained for both single-shade and conventional multi-shade nano-filled and bulk-fill composites. Regarding the outcomes, the incorporation of nanofillers improves the abrasive resistance, promoting a higher filler loading with consequent protection of the softer matrix, which reduces the interparticle spacing and simultaneously enhances the potential of the material to abrasive effect. After 12-month simulated toothpowder brushing, only bulk-fill composite presented Ra values below threshold of 0.2 µm (18) but they had not absolute value difference compared with the other composites.

This study had some limitations. First, it lacked accurate information regarding the compositions and physical and mechanical qualities of dentifrices and composites; the percentage of each component or whether any component was not listed could not be verified. This information is only available in scientific journals and only to a limited extent. In addition, laboratory tests did not simulate all oral environment conditions. Toothpowder usage-related factors associated with pH and temperature changes, brushing force, and occlusal wear could have damaged the resin composite, and these aspects should be addressed in future investigations.

However, the clinical relevance of the study is demonstrated by the finding that charcoal-based toothpastes do not influence color change, gloss, or surface roughness of intact or bleached enamel, single-shade, and multi-shade restorations after 12 months of toothbrushing compared to regular toothpaste. In contrast, charcoal toothpowder alters these parameters, compromising the longevity of restorations and compromising the morphological integrity of the enamel surface, particularly when previously bleached. Therefore, their use in oral hygiene is discouraged.

## Conclusions

Charcoal-based toothpaste impacts the morphology, roughness, gloss, and color of enamel and resin composites similarly to conventional toothpaste. However, toothpowder causes greater surface irregularities, leading to decreased gloss and increased color change and roughness, particularly affecting bleached enamel and composites more significantly after 12-months of simulated brushing.

## Figures and Tables

**Table 1:** Composition of materials used in this study (manufacturer data).

Material	Composition	Manufacturer
Colgate total 12 (Control)	Glycerin, aqua, hydrated Silica, Sodium Lauryl Sulfate, arginine, aroma, cellulose Gum, zinc oxide, poloxamer 407, zinc citrate, tetrasodium pyrophosphate, xanthan gum, benzyl alcohol, cocamidopropyl betaine, Sodium Fluoride (1500 ppm F−), sodium saccharin, and sucralose.	Colgate-Palmolive, São Paulo, SP, Brasil
Colgate Natural Extracts (Charcoal-based dentifrice - DC)	Aqua, glycerin, hydrated silica, sodium lauryl sulfate, aroma (peppermint oil), cellulose gum, xanthan gum, sodium fluoride (1450 ppm F−), sodium saccharin, charcoal powder, benzyl alcohol, eugenol.	
WhiteMax (Charcoal-based powder - PO)	Activated charcoal powder, Kaolin, Citrus aurantium dulcis peel oil, aroma.	Dermavita, Brusque, SC, Brasil
Nanohybrid composite (Opallis) (NH)	Active Ingredients: Bis-GMA (Bis-Phenol A di-Glycidyl Methacrylate), BisEMA (Bis-Phenol A di-Glycidyl Methacrylate) Monomers, TEGDMA (Triethylene Glycol Dimethacrylate), UDMA (Urethane Dimethacrylate), Camphorquinone, Co-initiator and silane. Inactive ingredients: silanized barium-aluminum silicate glass, pigments and silica. Shade A2. # 0.5 µm average particle size.	FGM, Dentscare LTDA, Joinville – SC, Brasil
Nano-filled (Vitra APS) (NF)	Active ingredients: monomeric matrix containing UDMA (Urethane Dimethacrylate) and TEGDMA (Triethylene Glycol Dimethacrylate) type monomers, photoinitiator composition (APS), co-initiators, stabilizer and silane. Inactive ingredients: zirconia filler, silica and pigments. Shade A2. # 0.2 µm average particle size.	
Single-shade nano-filled composite (Unique - SS)	Active ingredients: mixture of methacrylate monomers, photoinitiator composition (APS), co-initiators, stabilizers and silane. Inactive Ingredients: barium-aluminium-silicate glass. # 0.2 µm average particle size.	
Nanohybrid bulk fill (Opus Bulk fill) (BF)	Active Ingredients: Urethanedimethacrylic monomers, stabilizers, photoinitiators and co-initiators. Inactive Ingredients: Inorganic fillers of silanized silicon dioxide (silica), stabilizers and pigments. Shade A2. # 0.8 µm average particle size.	
Whiteness HP Blue Calcium (Bleaching product)	After mixing the phases: 35% hydrogen peroxide, thickeners, neutralizer, calcium gluconate, glycol, inert blue or violet dye, deionized water. Protocols of use: 2 applications for 40 minutes.	

**Table 2:** Means (±standard deviation) of composite resin ΔE00 and gloss comparing brushing times to products (n=11).

	COLOR CHANGE									GLOSS UNIT			
Composite resin	Brushing products	Assessment time								Assessment time			
		T1		T2		T6		T12		Baseline		After brushing	
NH	Control	0.87±0.4	Aa	0.90±0.5	Aa	1.01±0.5	Aa	0.65±0.3	Aa	63.0±1.8	Aa	31.1±3.3	Ab
	PO	0.74±0.4	Aa	1.47±6.0	Bb	2.83±1.0	Bb*	2.30±0.6	Bb*	63.0±1.8	Aa	23.9±4.1	Bb
	DC	0.84±0.3	Aa	1.19±0.3	Aa	1.07±0.6	Aa	1.08±0.4	Aa	62.7±2.6	Aa	32.9±3.3	Ab
NF	Control	0.66±0.5	Aa	0.53±0.2	Aa	0.77±0.5	Aa	0.75±0.3	Aa	64.3±4.7	Aa	30.6±4.2	Ab
	PO	1.40±0.9	Aa	1.73±1.0	Bb	2.64±1.1	Bb*	3.05±1.0	Bb*	64.2±4.6	Aa	20.5±2.2	Bb
	DC	0.76±0.7	Aa	0.87±0.6	Aa	0.97±0.6	Aa	0.93±0.4	Aa	64.0±4.6	Aa	28.4±5.0	Ab
SS	Control	0.86±0.5	Aa	0.77±0.5	Aa	0.88±0.5	Aa	0.89±0.5	Aa	53.8±3.8	Aa	14.7±1.6	Ab
	PO	0.54±0.3	Aa	1.27±0.6	Bb	2.22±1.1	Bb*	2.67±1.0	Bb*	53.0±4.6	Aa	15.4±1.5	Ab
	DC	0.59±0.4	Aa	0.60±0.4	Aa	0.73±0.4	Aa	0.83±0.6	Aa	52.9±4.7	Aa	15.1±1.8	Ab
BF	Control	0.63±0.5	Aa	0.85±0.5	Aa	0.90±0.6	Aa	1.06±1.1	Aa	40.2±3.7	Aa	17.3±2.4	Ab
	PO	0.59±0.4	Aa	0.87±0.7	Bb	1.78±0.8	Bb	2.30±0.9	Bb*	40.2±3.7	Aa	18.4±8.0	Ab
	DC	0.52±0.4	Aa	0.53±0.7	Aa	0.74±1.2	Aa	0.90±1.1	Aa	39.9±3.8	Aa	17.6±2.5	Ab

Different letters (lowercase for comparing the brushing times - in line; uppercase for comparing brushing products – in columns) indicate significant differences for each optical property at Tukey`s test (*P*<0.05). * Color changes beyond the acceptability threshold (ΔE00=1.77)7.

**Table 3:** Means (±standard deviation) of ΔE00 and gloss for intact and bleached enamel comparing brushing times to products (n=22).

COLOR CHANGE						GLOSS UNIT	
Enamel	Brushing products	Assessment time					
		T1	T2	T6	T12	Baseline	After brushing
Intact	Control	1.67±1.11	1.44±1.00	1.12±1.06	1.32±0.92	98.2±1.5 Aa	95.7±4.6 Aa
	PO	0.65±0.44	0.52±0.27	0.55±0.36	0.74±0.36	97.5±1.2 Aa	81.2±7.6 Bb
	DC	1.04±0.59	1.03±0.69	1.30±0.88	1.03±0.58	98.4±1.9 Aa	53.1±5.3 Cb
Bleached	Control	0.57±0.44	0.59±0.42	0.71±0.33	0.70±0.36	97.9±2.5 Aa	96.9±4.7 Ab
	PO	0.98±0.66	1.05±0.62	1.34±1.00	1.08±0.58	98.2±1.9 Aa	62.1±7.1 Bb*
	DC	0.81±0.52	1.01±0.77	1.11±0.80	1.14±0.60	97.1±2.2 Aa	56.4±4.4 Bb

Repeated measured ANOVA indicated no differences between groups for ΔE00 (*p*<0.05). Different letters (lowercase for comparing assessment time - in line; uppercase for comparing brushing products – in columns) and symbol (*) for comparing enamel gloss in the same dentifrice and assessment time indicate significant differences at Tukey`s test (*P*<0.05).

**Table 4:** Means (±standard deviation) of roughness average (Ra) for intact and bleached enamel comparing brushing times to products.

Enamel	Brushing products	Assessment time				
		T0	T1	T2	T6	T12
Intact	Control	0.02±0.01^ Aa^	0.02±0.01^ Aa^	0.03±0.02^ Aa^	0.03±0.02^ Aa^	0.04±0.04^ Aa^
	PO	0.03±0.01^ Aa^	0.03±0.01 ^Ab^	0.03±0.01^Ac^	0.05±0.02^Ac^	0.08±0.03^Bd^
	DC	0.02±0.01^ Aa^	0.03±0.01^ Aa^	0.03±0.01 ^Aab^	0.03±0.01^Ab^	0.05±0.02^Ab^
Bleached	Control	0.02±0.01^ Aa^	0.03±0.01^Aa^	0.03±0.01^ Aa^	0.03±0.30^ Aa^	0.03±0.01^Aa^
	PO	0.02±0.01^ Aa^	0.03±0.01^Ab^	0.05±0.03 ^Ac^^*^	0.07±0.04^Ac^^*^	0.10±0.05^Bd^^*^
	DC	0.03±0.01^ Aa^	0.03±0.01^ Aa^	0.04±0.01 ^Aab^	0.06±0.02^Ab^^*^	0.06±0.03^Ab^^*^

Brushing times: T1- baseline, T1- 1-month, T2- 2-months, T6- 6-months, and T12- 12-months. Control: Colgate Total 12 (Colgate-Palmolive); PO- Activated carbon powder - WhiteMax (Dermavita) and DC- activated carbon-based toothpaste - Colgate Natural Extracts (Colgate-Palmolive). Different letters (lowercase for comparing the brushing times - in line; uppercase for comparing brushing products – in columns) indicate significant difference at Tukey`s test (*P*<0.05) and symbol (*) for comparing enamel indicate significant differences at Tukey`s test (*P*<0.05).

## Data Availability

The datasets used and/or analyzed during the current study are available from the corresponding author.
